# The posterior approach reduces the risk of thin cement mantles with a straight femoral stem design

**DOI:** 10.3109/17453674.2010.487239

**Published:** 2010-05-21

**Authors:** Gavin J Macpherson, Christian Hank, Michael Schneider, Morag Trayner, Robert Elton, Colin R Howie, Steffen J Breusch

**Affiliations:** Department of Orthopaedic Surgery, University of Edinburgh, The Royal Infirmary of EdinburghUK

## Abstract

**Background and purpose:**

The properties of the cement mantle around a prosthesis are important. We investigated whether the surgical approach to the hip influences the quality and thickness of the cement mantle when using a straight femoral stem design.

**Methods:**

In a consecutive multi-surgeon series, we reviewed the radiographs of 270 patients after cemented Exeter total hip arthroplasty. 135 stems were introduced using an antero-lateral (transgluteal) approach and 135 stems were introduced using a posterior approach. Anterior-posterior and lateral radiographs were reviewed and cement mantle thickness was measured in Gruen zones 1–14. We graded cement mantle quality according to the Barrack classification.

**Results:**

Barrack grading did not reveal any difference in cement mantle quality between the two groups. AP and lateral radiographs showed no difference in stem alignment between the groups. The risk of a thin cement mantle (< 2 mm) was lower with a posterior approach (OR = 1.8, 95% CI: 1–3; p = 0.03). The greatest risk of a cement mantle thickness of < 2 mm occurred in Gruen zones 8–9 regardless of the surgical approach used.

**Interpretation:**

With a straight femoral stem design, the posterior approach to the hip joint appears to give a lower risk of a thin cement mantle. Irrespective of the approach, there was a risk of thin cement mantles in Gruen zones 8 and 9, which highlights the importance of lateral radiographs in the postoperative radiographic assessment of total hip replacements.

## Introduction

There is no data available that defines the optimal cement mantle thickness in total hip arthroplasty. There is, however, evidence that a cement mantle of 2–3 mm is associated with a better long-term radiographic outcome (Ebramzadeh et al. 1994, Joshi et al. 1998). Thin cement mantles are more prone to crack (Kwak et al. 1979, Kawate et al. 1998), thus opening the bone-cement interface to wear particles and fluid pressure (Bartlett et al. 2008). This has been documented to cause localized osteolysis (Huddleston 1988, Anthony et al. 1990, Schmalzried et al. 1997) and failure (Olsson and Jernberger 1981, Jasty et al 1991, Cristofolini et al. 2007). Several factors influence the quality of the cement mantle: the cementing technique (Mulroy et al. 1990, Britton et al. 1996, Malchau and Herberts 1998), femoral anatomy (Crawford et al. 1999, Breusch et al. 2001), stem design and instrumentation (Garellick et al. 1999, Östgaard et al. 2001), centralizer usage (Berger et al. 1997), and stem size (Krismer et al. 1991, Massoud et al. 1997, Scheerlinck et al. 2008).

Due to the anatomical shape of the proximal femur, there is a risk of producing thin cement mantles anterior-proximally (Garellick et al. 1999, Breusch et al. 2001, Valdivia et al. 2001). An antero-lateral surgical approach has been shown to adversely affect the orientation of the tip of the femoral component (Vaughan et al. 2007), but little else is known about the effect of the surgical approach to the hip with regard to stem alignment and cement mantle thickness or quality.

We investigated the influence of surgical approach on cement mantle thickness and cement mantle quality using the straight Exeter femoral stem by comparison of posterior and antero-lateral approach in a multi-surgeon series.

## Patients and methods

This was a prospective, consecutive, multi-surgeon series of 270 cemented primary total hip replacements performed in 2005 at our university teaching hospital (Table 1). Patients with a previous femoral osteotomy were not included. The Exeter stem (Stryker Orthopaedics, Mahwah, NJ) was used in all cases. All procedures were carried out with the patient in a lateral decubitus position. 135 patients were operated on using an antero-lateral approach to the hip joint and 135 patients using a posterior approach.

**Table 1.** Demographic data of study group

	Anterolateral approach (n = 135)	Posterior approach (n = 135)
Patients (bilateral)	115 (20)	122 (13)
Mean age (range)	67 (31–90)	68 (46–82)
Male/female	38/97	51/84
Left/right	68/67	68/67
Diagnosis		
OA	120 (89%)	114 (85%)
RA	0	2 (2%)
Post-traumatic OA	2 (2%)	3 (2%)
DDH	7 (5%)	10 (8%)
Other	6 (5%)	6 (5%)
BMI (range)	29 (17–34)	28 (21–32)

BMI only available for 204 patients: 101 patients in the antero-lateral group, 103 patients in the posterior group.

Preoperative templating on AP radiographs was undertaken to determine the optimum femoral implant allowing for a cement mantle of at least 2 mm. All operations were performed with third-generation cementing techniques (distal femoral cement restrictor, pulsatile lavage, retrograde cement application via gun, and cement pressurization with a proximal femoral silicon seal). All procedures were carried out either by a consultant arthroplasty surgeon or a trainee under the direct supervision of a consultant arthroplasty surgeon. Our practice is to remove the prominent posterior calcar femorale to permit posterior canal entry. The stem size corresponding to the last broach inserted allowed for a nominal minimal cement mantle of 2 mm.

Radiographs with standard anterior-posterior (AP) views were taken 2 days after surgery. No lateral views were obtained at this stage, due to difficulties in positioning the patient in the early postoperative period. Lateral radiographs were obtained 1 year postoperatively at routine follow-up.

Radiographic analysis was done by CH and MS, who were not involved with the surgical procedures. The analysis included comparison of early postoperative AP radiographs and AP and lateral radiographs taken 1 year postoperatively. The measurements were taken with a calibrated sliding ruler using the femoral head size as a reference.

The integrity of the cement mantle was graded according to Barrack et al. (1992) and cement mantle thickness was measured in all 14 Gruen zones. Varus/valgus and lateral malalignment of the stem was defined as a deviation from the longitudinal axis of 3 or more degrees (Joshi et al. 1998). The femoral diaphysis was used as the reference on the lateral radiographs.

### Statistics

Chi-squared tests using SPSS version 12.0 software were used for unadjusted comparisons between the two groups, and mixed-model logistic regression using MLwiN software was used to test the effect of approach after adjusting for the potential confounding effects of age, BMI, sex, diagnosis, stem alignment on AP, and lateral views. This method also allowed for the lack of independence of results from patients undergoing bilateral operations.

## Results

### Cement mantle quality (Table 2)

Chi-squared testing did not reveal any statistically significant differences in cement mantle quality between the groups (p = 0.1).

**Table 2.** Cement mantle integrity according to Barrack

Barrack classification	Antero-lateral n (%)	Posterior n (%)
A	40 (30)	44 (33)
B	93 (69)	84 (62)
C	2 (2)	7 (5)
D	0	0

### Stem alignment (Tables 3 and 4)

There was no statistically significant difference in stem alignment between the two groups on AP (p = 0.06) and lateral radiographs (p = 0.5).

**Table 3.** Stem alignment on AP radiograph

Alignment	Antero-lateral n (%)	Posterior n (%)
Neutral	121 (90)	111 (82)
Varus	7 (5)	21 (16)
Valgus	7 (5)	3 (3)

**Table 4.** Stem alignment on lateral radiograph

Alignment	Antero-lateral n(%)	Posterior n (%)
Neutral	104 (77)	102 (76)
Anterior	5 (4)	19 (14)
Posterior	26 (19)	14 (10)

### Cement mantle thickness (Table 5)

In the antero-lateral approach group, only 47% had a cement mantle of at least 2 mm in all 14 zones, compared to 62% in the posterior approach group. This difference was statistically significant (OR = 1.8, 95% CI: 1.1–3.0; p = 0.03). After adjusting for age, sex, diagnosis, cement mantle quality, and stem alignment, the surgical approach remained significant (OR = 2.5, 95% CI: 1.4–4.6; p = 0.002). The highest incidence of reduced cement mantle thickness in both groups was seen antero-proximally on lateral radiographs (Gruen zones 8 and 9).

**Table 5.** Cement mantle thickness on AP and lateral radiographs. Values are percentage with antero-lateral/posterior approach

Gruen zone														
	1	2	3	4	5	6	7	8	9	10	11	12	13	14
≥ 2 mm	98/99	98/99	98/99	98/99	97/97	96/95	99/99	68/79	72/81	99/99	99/99	97/97	100/100	99/99
< 2 mm	2/1	2/1	2/1	0/0	2/2	3/3	1/1	27/19	26/15	1/1	0/0	2/3	0/0	1/1
< 1 mm	0/0	0/0	0/0	2/1	1/1	1/2	0/0	5/2	2/4	0/0	1/1	1/0	0/0	0/0

In the antero-lateral approach group, only 68% had a cement mantle thickness of ≥ 2 mm in Gruen zone 8. In the posterior approach group, 79% had a ≥ 2-mm cement mantle in the same Gruen zone. In Gruen zone 9, 72% in the antero-lateral group and 81% in the posterior approach group had a cement mantle thickness of ≥ 2 mm. In Gruen zone 12 (postero-distally), only 3% in the antero-lateral approach group and 3% in the posterior approach group had a cement mantle thickness of < 2 mm.

## Discussion

Evaluation of the cement mantle is an important tool for quality and outcome assessment of cemented total hip arthroplasty. Poor cement mantle quality has been identified as a predictor of worse long-term outcome (Chambers et al. 2001). Most previous studies have only assessed the cement mantle on AP radiographs. It is well documented that if true lateral radiographs are not taken, then the risk of thin cement mantles is underestimated (Breusch et al 2001, Östgaard et al 2001). Östgaard et al. (2001) observed a similar pattern of malaligned Charnley stems in the lateral plane as we did, confirming previous reports (Crawford et al. 1999, Breusch et al 2001, Valdivia 2001). In a historical comparison to an original Charnley series, lateral cement mantles were less deficient in cases with trochanteric osteotomy (Garellick et al. 1999). A similar lateral stem malalignment pattern was found with other stem designs and stem-bone contact was noted in Gruen zone 8 in 20% of cases with Charnley stems and 13% with the Spectron stem (Garellick et al 1999). In this context, lower femoral neck osteotomies and more aggressive removal of the posterior femoral neck (Wroblewski et al. 2000) were advocated. This allows a more posterior entry point, and as a consequence the alignment of a straight stem design is improved. Vaughan et al. (2007) reported a tendency for lateral stem malalignment and posterior stem tip-to-cortex contact, as was also observed in the present study.

Little is known regarding the influence of the surgical approach to the hip on the cement mantle, aseptic loosening, and overall revision rates. RSA data have shown a higher incidence of rotational instability with the Exeter stem using the posterior approach than when using the lateral approach (Glyn-Jones et al. 2006).

Our results suggest that a posterior approach is associated with a lower risk of thin cement mantles. We can only hypothesize that exposure of the fossa piriformis and canal entry may be easier with a posterior approach. When using an antero-lateral approach in obese or very muscular patients, the broach and stem have a tendency to be pushed anteriorly by the soft tissue, thus providing an explanation for sagittal stem malalignment. In our series, however, a high BMI was not predictive in this context.

An efficient distal centralizer appears to eliminate the problem of a distal stem tip-to-cortex contact and its detrimental effect in zone 12 (Berger et al. 1997, Garellick et al. 1999, Breusch et al. 2001), and our findings support this also. However, it has been shown that a distal centralizer cannot prevent thin cement mantles in zone 8/9 (Breusch et al. 2001). This has been confirmed in our study, where the distal centralizer did not protect from a thin cement mantle anteriorly in approximately a quarter of the cases. These findings are also in keeping with the results of cadaver studies with Exeter stems (Valdivia et al. 2001, Mayr et al. 2006).

The clinical relevance of these thin cement mantles is subject to debate. It is clear from the published outcome data that a thin cement mantle anterior-proximally is a frequent finding around Exeter and other straight stems, but with polished, tapered designs this does not seem to adversely affect outcome in the first decade (Berli et al. 2005, Hook et al. 2006, Lewthwaite et al. 2008). In the second decade, when more wear particles may have accumulated, osteolysis is more common and rates of femoral osteolysis from 32–70% have been described (Räber et al 2001, Iwase et al. 2002). Osteolysis-induced periprosthetic fracture then becomes a more frequent failure mechanism (Lindahl et al. 2005). In the presence of polyethylene wear, thin cement mantles may play a future role in failure during the second decade (Kerboull et al. 2004). Stems designed to be implanted with thin cement mantles (line to line) have shown excellent survival after a mean of 14 years (Kerboull et al. 2004). However, the cases that had failed due to aseptic loosening/osteolysis were all in a high wear rate group, thus emphasizing the importance of a non-deficient cement mantle. In this biological context, the integrity of the cement mantle becomes most important. Open access of the bone interface, via mantle defects, to particles and fluid pressure has been identified as an important factor for osteolysis (Schmalzried et al. 1997, Bartlett et al. 2008).

Although we do not know the long-term implications of our findings, the effect on the cement mantle should also be considered when choosing the approach to the hip. It seems reasonable to favor a posterior approach in younger patients, who are likely to survive the first decade after implantation. In older patients, other factors may play a more important role when choosing the surgical approach. Regardless of the approach, meticulous bone preparation with posterior canal entry remain important operative steps to overcome the dilemma of achieving a non-deficient cement mantle with straight stem designs in the curved proximal femur.
